# One-year Follow-up Results of Transperineal Biopsy For Patients Undergoing Irreversible Electroporation Treatment in Localized Prostate Cancer

**DOI:** 10.5152/tud.2023.23150

**Published:** 2023-11-01

**Authors:** Şükrü Ali Altan, Pınar Güleryüz Kızıl, Nefise Çağla Tarhan, Oztug Adsan

**Affiliations:** 1 Department of Urology, TOBB University of Economics and Technology, Faculty of Medicine, Ankara, Turkey; 2 Department of Radiology, TOBB University of Economics and Technology, Faculty of Medicine, Ankara, Turkey; 3 Department of Radiology, Acıbadem University, Faculty of Medicine, Istanbul, Turkey

**Keywords:** Irreversible electroporation, focal ablation, follow-up, prostate cancer, treatment

## Abstract

**Objective:**

This article reports on the early results of a 1-year follow-up study investigating the efficacy of irreversible electroporation in the treatment of localized prostate cancer.

**Methods:**

The study included 18 out of 40 patients diagnosed with low- and intermediate-risk prostate cancer who underwent irreversible electroporation. Treatment results were evaluated through confirmation biopsies, comparing prostate-specific antigen levels, international prostate symptom scoring, and international index of erectile dysfunction scores before irreversible electroporation and at the 12-month mark.

**Results:**

The mean age of the patients was 61.1 years (SD ±6.5). Out of the 18 patients, 16 were tumor free (88.8%), while 2 experienced recurrences, one within the treatment field and the other outside of it (*P* < .001). Irreversible electroporation significantly reduced mean prostate-specific antigen levels (6.73 ng/mL vs. 2.05 ng/mL, *P* < .001), indicating a 69.5% reduction within 12 months. Furthermore, there was a significant improvement in mean international prostate symptom scores at the 12-month follow-up (10.05 vs. 7.52, *P* = .003). The mean international index of erectile dysfunction scores before treatment was 19.17 (SD ±5.85), and after irreversible electroporation, it was 18.67 (SD ±6.34), with no statistically significant change (*P* = .065).

**Conclusion:**

The short-term oncological results of irreversible electroporation treatment are promising, particularly for patients in the low- and intermediate-risk groups. Additionally, irreversible electroporation does not negatively impact the international index of erectile dysfunction; however, it may lead to a decrease in international prostate symptom scores.

**Main Points**

- It has been shown that the irreversible electroporation method used in the focal treatment of prostate cancer has a low incidence of side effects.
- It has been shown that an effective oncological response was obtained in the 1-year follow-up of focal treatment with irreversible electroporation method in low- and intermediate-risk prostate cancer patients.
  Irreversible electroporation therapy has been shown to be an effective method in the focal treatment of low and medium risk prostate cancer patients.

## Introduction

Currently, radical prostatectomy and radiotherapy are the most commonly used treatment methods for early-stage prostate cancer. However, these treatments often result in severe side effects, such as erectile dysfunction and urinary incontinence, which significantly impact the patients' quality of life.^1^ In recent years, there has been a growing interest in focal treatments for prostate cancer, as they have shown comparable oncological outcomes while significantly reducing side effects and improving the quality of life.^2^

Various methods and energy sources, including irreversible electroporation (IRE), high-intensity focused ultrasound, cryoablation, laser ablation, transurethral ultrasound ablation, low-dose brachytherapy, and photodynamic therapy, are being explored for the focal treatment of localized prostate cancer.^3^ Among these methods, IRE offers a significant advantage. It is a non-thermal focal ablation technique that does not cause thermal damage to the surrounding soft tissues and anatomical structures near the tumor.^4^

Initial studies conducted on localized prostate cancer patients have demonstrated low patient morbidity and favorable short-term oncological control with IRE treatment.^5-11^ Consequently, IRE treatment appears to be a promising focal treatment option for low- and intermediate-risk patients.^8^ However, there is a need to further investigate the oncological results of this therapy, particularly in these risk groups. Therefore, the objective of this study is to assess the treatment efficacy and quality of life of patients who have completed 1 year of follow-up after undergoing IRE treatment, as confirmed by biopsy results.

## Material and Methods

Eighteen out of 40 patients diagnosed with prostate cancer by fusion biopsy since November 2020 and subsequently treated with IRE were included in this study. The inclusion criteria for the study were patients who had first-year confirmation biopsies and were defined as low- and intermediate-risk patients with localized prostate cancer according to the guidelines of the European Urology Association. Patients in the high-risk group and those who had not yet undergone their first-year confirmation biopsies were excluded from the study. Lesions not visible on MRI and patients diagnosed only on systematic biopsy were not included in this study. None of the patients in the study received any additional treatment, such as androgen-deprivation therapy.

For the clinical staging of the patients, multiparametric prostate magnetic resonance imaging (mpMRI) and/or prostate-specific membrane antigen positron emission tomography (PSMA PET) were utilized. Irreversible electroporation was administered to 1 field in 14 patients and to 2 fields in 4 patients. Follow-up assessments were conducted every 3 months through prostate-specific antigen (PSA) levels and clinical interviews, every 6 months through mpMRI, and at the 12-month mark through confirmation fusion biopsy.

### Multiparametric Prostate Magnetic Resonance Imaging and Image-Fusion Biopsy

A 3T MRI system (Ingenia, Philips Medical System, Netherlands) was used for screening all patients. MpMRI was employed for the 12-month follow-up of patients who underwent focal ablation with IRE. These images were compared with pre-procedure mpMRI and evaluated according to the criteria set by the Prostate Imaging and Reporting Data System (PI-RADS) Steering Committee (version 2.1). Changes resulting from the treatment, potential residual malignancies, and lesions indicating malignancies outside the treatment area were reported separately by comparing the examinations with previous mpMRIs. The mp-MRI images were assessed by experienced radiologists.

**Figure 1.**
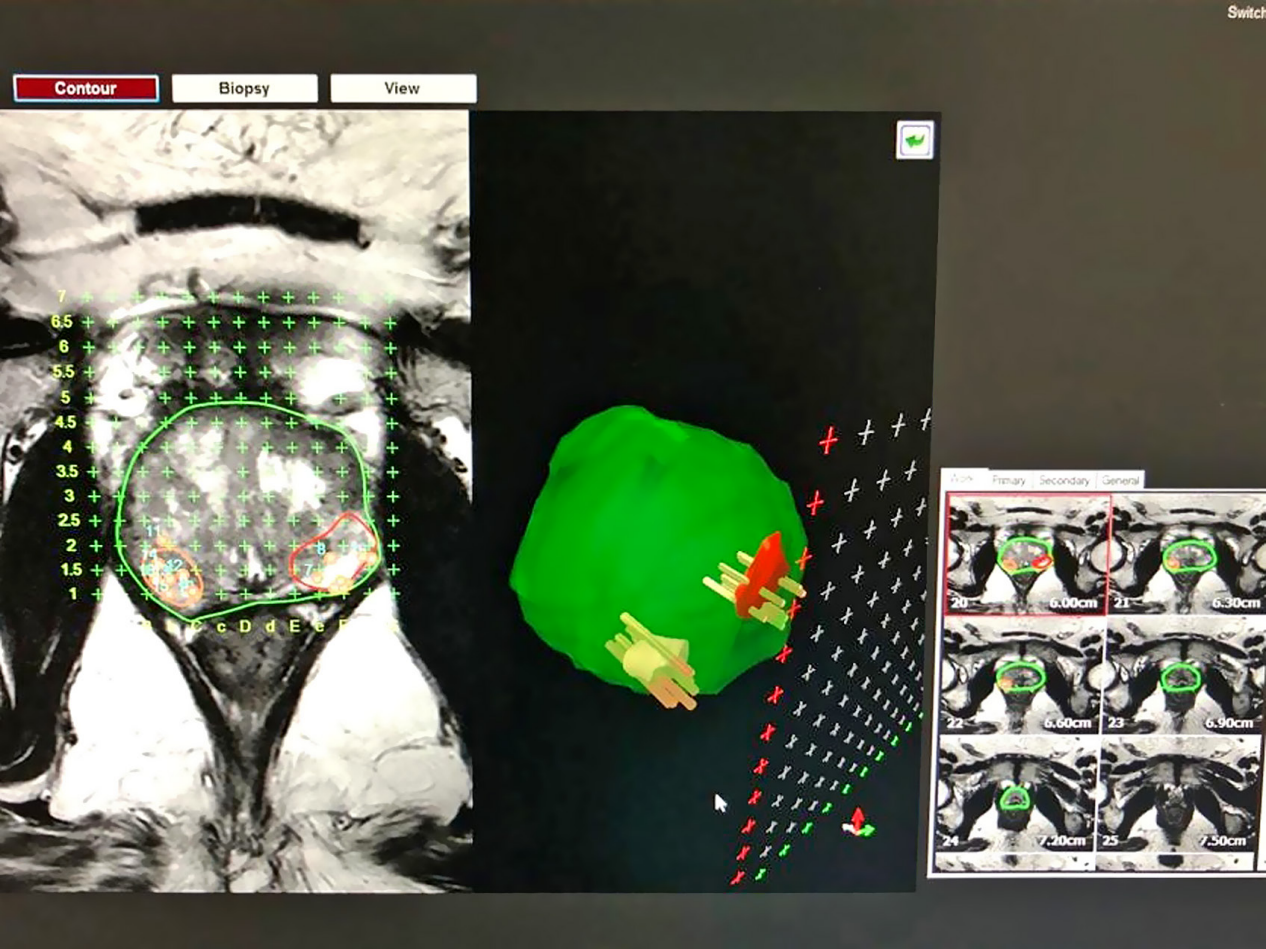
Three-dimensional view of targeted transperineal fusion biopsy areas.

Following mpMRI, a targeted transperineal fusion biopsy protocol was implemented using the Bioject TM software (D&K Technologies, Barum, Germany) for prostate mapping (see Figure 1). This transperineal biopsy technique was performed on the patients. Pathology samples were evaluated by an experienced pathologist. The results of the follow-up biopsies were reported as negative, malignancy in the treatment field, or malignancy outside the treatment field

### Irreversible Electroporation Method

**Figure 2.**
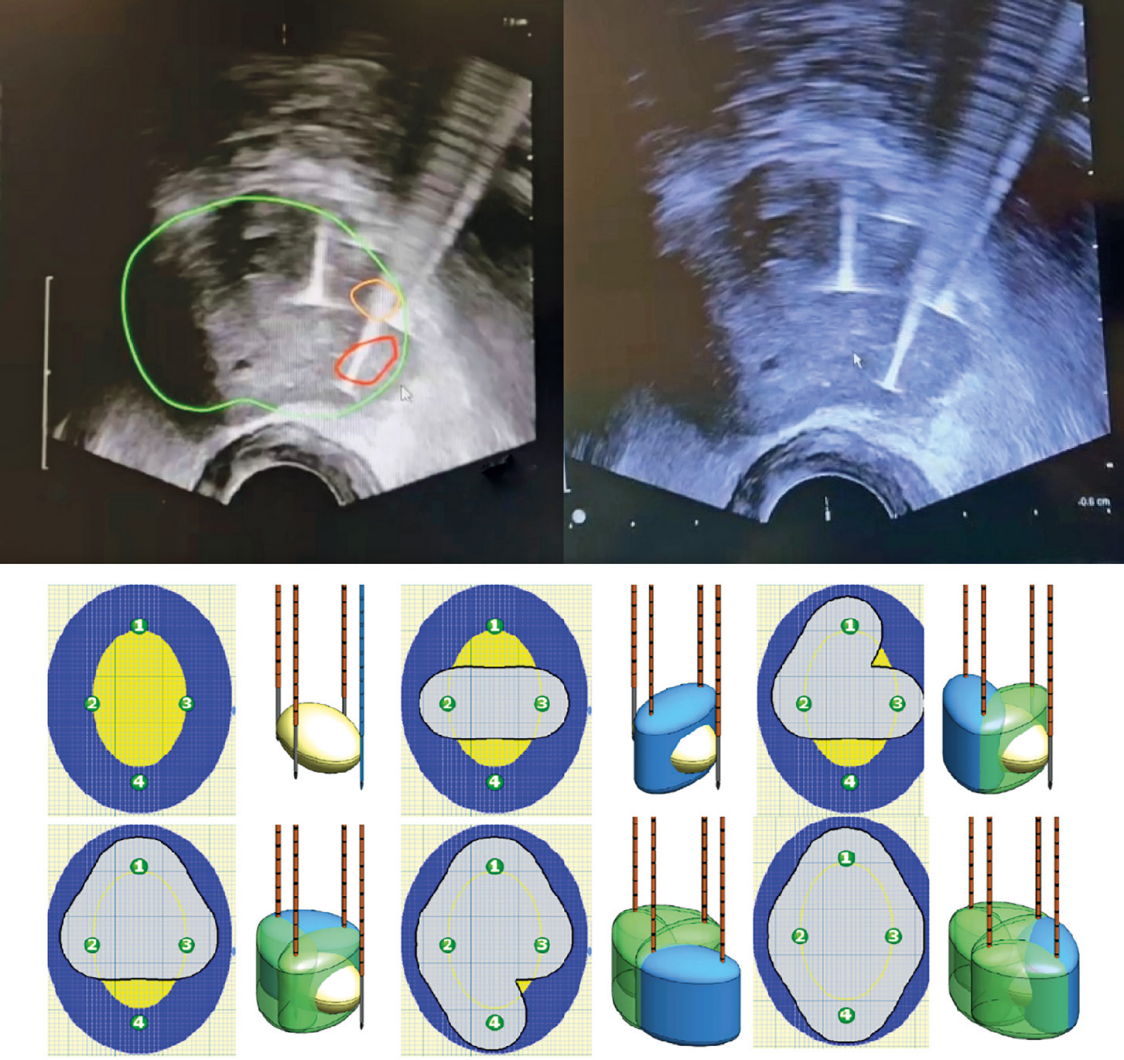
Schematic and real-time application of irreversible electroporation needles.

All patients underwent the procedure under general anesthesia with deep muscle relaxation while positioned in a lithotomy. A urethral catheter was inserted for bladder drainage. A transrectal biplanar ultrasound (BK Medical, Herlev, Denmark) probe and a transperineal template were utilized. Three or 4 IRE electrodes were strategically placed around the previously mapped lesion. A 5 mm safety distance from the prostatic capsule to protect the neurovascular bundle and rectum. The distance between the active electrodes ranged from 1 to 2 cm, and the depth of the electrodes was measured using biplanar ultrasound and recorded in the IRE system (Nanoknife, angio-Dynamics, NY, USA). Initially, a test flow was administered to assess the optimal flow level. Subsequently, the treatment dose was administered (see Figure 2). In patients treated for a single lesion, the urethral catheter was removed on the day after the procedure, while patients treated in 2 fields or a larger field had the catheter removed after 5 days. Alpha blocker treatment was administered to the patients for 1 month following the IRE procedure.

### Quality of Life and Functional Questioning

Quality of Life and Functional Questioning: The patients included in the study were assessed using the International Prostate Symptom Score (IPSS) and International Index of Erectile Dysfunction (IIEF) before undergoing IRE. These evaluations were repeated every 3 months. In this study, we compared the IPSS and IIEF results before IRE with the results obtained at the 12-month mark.

Ethics Committee approval of the research protocol by an institutional review board was obtained from TOBB University (Protocol Number of Ethics Committee Approval: TOBB ETU KAEK-118/096). All patients were given information about the procedure, and their consent were obtained.

### Statistical Analysis

The statistical analysis was performed using the Statistical Package for Social Sciences Statistics software for Windows, version 17.0 (SPSS Inc.; Chicago, IL, USA). Descriptive statistics, such as frequencies, means, and SDs, were calculated. Categorical variables were presented as percentages. The normal distribution compatibility of the variables was tested using analytical methods such as the Kolmogorov–Smirnov or Shapiro–Wilk tests. The Wilcoxon signed-rank test and paired samples test were used to compare 2 related samples, depending on the normality of their distribution, in order to assess whether their population mean ranks differed.

## Results

### Patient Characteristics

The mean age of the patients was 61.1 (SD ±6.5) years. The average PSA level before IRE treatment was 6.73 ng/mL (SD ±2.98 ng/mL). Regarding the treated fields, 7 were diagnosed as International Society of Urologic Pathologists (ISUP) grade 1 prostate cancer, 6 as ISUP grade 2, and 5 as ISUP grade 3. Among the 22 treated fields, 21 had cancer located in the peripheral zone, while only 1 patient had a lesion in the transitional zone. Eight treatment fields were located at the apex, 8 in the midgland, and 6 at the base. The mean diameter of the lesions was 11.8 mm (SD ±7.4 mm). Further details of patient characteristics are summarized in Table 1.

### Treatment Results

At 12 months, control transperineal fusion biopsies were performed on the lesion areas as well as systematically randomized areas following the IRE procedure. Among the 18 treated patients, 16 were found to be tumor free, while 2 showed recurrences in the control fusion biopsies (*P* < .001). One recurrence was observed within the treatment field, and a new tumor development was detected at a different location outside the IRE field. The patient with recurrence in the treatment area underwent a second session of IRE, resulting in no recurrence during the first-year follow-up. However, the patient with a new tumor development outside the treatment area declined a second IRE session. Both patients who experienced recurrence were classified in the intermediate-risk group for recurrence.

A magnetic resonance examination performed before the control transperineal fusion biopsy revealed that 3 of 18 patients were positive for residual tumors. Two of these 3 patients were found to have residual tumors pathologically, while 1 case was reported as benign.

The mean PSA level before IRE was 6.73 ng/mL (SD ±2.98 ng/mL), and after 12 months, the PSA level decreased to 2.05 ng/mL (SD ±1.26 ng/mL) (*P* < .001). The PSA levels showed a significant decrease of 69.5% within the 12-month period in all patients.

### Quality of Life and Functional Questioning Results

The mean IPSS score for the study participants was 10.05 (SD ±7.02), and after 12 months of treatment, it significantly improved to 7.52 (SD ±4.71) (*P* = .003). The mean IIEF scores before IRE treatment were 19.17 (SD ±5.85), and following irreversible electroporation, the scores were 18.67 (SD ±6.34). There was no statistically significant change in the mean IIEF scores (*P* = .065).

## Discussion

Since November 2020, we have been using IRE techniques in our clinic as a standard approach for the focal treatment of prostate cancer. We obtained radiologically convincing results during the first 6-month follow-up of our patients and published our findings.^12^ While mpMRI is considered an important diagnostic tool for evaluating patient outcomes, obtaining biopsies from the treatment field, as well as standard random biopsies, remains the most crucial criterion for assessing treatment efficacy.

In the literature, studies with large patient populations have shown promising outcomes of IRE treatment in low- and intermediate-risk prostate cancer patients, based on long-term controlled oncological results. Specifically, intermediate-risk patients have demonstrated greater benefits from this approach.^7,8,11,13^ In our study, we carefully selected low- and intermediate-risk groups to ensure homogeneous treatment results. In low- and medium-risk prostate cancer patients, recurrence rates within the treatment field at 6- and 12-month follow-ups ranged from 3% to 16.7%.^7-11,13-17^ Gielchinsky et al^17^ conducted 1 of the recent studies on IRE treatment for prostate cancer, involving 45 patients, and reported recurrence rates of 4% within the treatment field and 12% outside the treatment field 1 year after IRE treatment. Their study included not only low- or intermediate-risk groups but also salvage group patients, which could have affected the recurrence rates within and outside the treatment field. In contrast, our study specifically focused on low- and intermediate-risk groups to achieve more homogenous treatment results.

When it comes to the follow-up of focal treatments, obtaining biopsies from the treatment field is considered the gold standard. For IRE treatments, where a 5 mm margin is left based on MR images, the risk of detecting clinically significant cancer in biopsies obtained from the treatment field is lower than 10%.^7-11,13^ While some studies evaluate recurrence rates based on clinically significant cancer definitions, the most comprehensive approach would be to consider the overall cancer rate. In our study, among low- and medium-risk cancer cases, the recurrence rate within the treatment field was 5.5% (1/18). In 1 patient, recurrence was identified within the treatment field, which had a relatively larger size of 15 mm. Another patient experienced a recurrence with ISUP grade 1 prostate cancer diagnosed through a randomized biopsy outside the treatment area, while no tumor was observed within the treatment area. Although the patient was offered a second session of IRE, he decided to undergo a radical prostatectomy.

A major counterargument against focal treatment of prostate cancer is the multifocality of the disease. Biopsies obtained from outside the treatment field often reveal low-risk prostate cancer. However, treating the index lesion, which is defined as the primary lesion, can effectively halt the progression of the disease. Therefore, by targeting the index lesion and any other visible lesions on MRI, the objective of focal treatment can be achieved. Recent studies conducted with this principle have demonstrated that treating index lesions prevents recurrence or the development of new lesions.^7-9,13-15,18^

The recommended follow-up protocol after focal treatment of prostate cancer should span a minimum of 2 years, including PSA measurements every 3 months, mpMRI every 6 months, and annual biopsies.^16^ Changes in PSA values are generally influenced by prostate size and the presence of other diseases, but on average, there is an approximate 70% decrease in PSA levels following treatment. In our series, the mean PSA decrease was 69.5% within 12 months.

For quality of life and clinical assessment, we employed IIEF and IPSS surveys. After 12 months of follow-up, we did not observe any significant difference in IIEF scores, indicating sexual function, while there was a statistically significant improvement in IPSS scores, reflecting urinary symptoms. The fact that our patients did not report side effects such as urinary incontinence or erectile dysfunction has bolstered our confidence in the applicability of this treatment approach. In fact, the side effect rates of IRE treatment were found to be lower than those of alternative focal treatment methods in other published series.^2,3,8^

Although the first study on high-frequency IRE, a new application technique of IRE, has been published, we do not have any mature results on this method yet.^19^ Focal application of IRE should be the first choice, as it reduces the rate of side effects. However, studies have shown that while the same oncological results are achieved in patients undergoing hemiablation and even extended IRE, there is no significant difference between the side effect rates encountered.^20,21^ In recent years, with the increase in focal treatment options, whole gland treatments have started to be pushed to the background. Focal treatment options provide a great advantage over whole gland treatments, especially due to their low incidence of side effects. Early functional results are very good in focal treatments, and oncological results are also satisfactory in low- and intermediate-risk prostate cancer patients. Although the treatment logic of focal treatment options such as high-intensity focal ultrasound, cryotherapy, and focal laser ablation is mostly based on thermal damage, non-thermal energy source is only used in IRE. It has been reported in review articles discussing focal treatments that IRE has a very low incidence of side effects.^22,23^

Our study has several limitations, including a limited number of patients, a retrospective analysis, and only short-term results. We plan to achieve statistically stronger results and examine longer-term outcomes by including patients who underwent IRE but could not be included in the study due to a lack of time for confirmation biopsies in the first year.

In conclusion, we believe that IRE provides effective oncological control for prostate cancer patients with low ISUP grades. Short-term evaluations of side effects associated with IRE treatment offer hope for these patients, as it reduces the morbidity rates that may be experienced with radical therapies.

## Figures and Tables

**Table 1.** Characteristics of the Patients and Application Fields Undergoing Irreversible Electroporation

Number of patients	18
Low-risk group	5
Intermediate-risk group	13
Number of treatment fields per application	
Single field	14
Two fields	4
Age	
Mean	61.1 (SD ±6.5)
Positive rectal examination finding	5.5% (1/18)
Prostate volume (mL)	
Mean	35.4 (SD ±11.6)
Prostate-specific antigen (ng/mL)	
Mean	6.73 (SD ±2.98)
Side of the treatment fields	
Right	61.5% (8/13)
Left	38.5% (5/13)
PIRADS^†^ score of treatment fields	
3	11.1% (2/18)
4	50% (9/18)
5	38.9% (7/18)
ISUP^‡^ grade of treatment fields	
Grade 1	38.9% (7/18)
Grade 2	33.3% (6/18)
Grade 3	27.8% (5/18)
Localization of treatment fields	
Apex	36.4% (8/22)
Midgland	33.4% (8/22)
Base	27.2% (6/22)
Prostatic zone of treatment fields	
Peripheral zone	95.4% (21/22)
Anterior fibromuscular stroma	4.6% (1/22)
Diameter of treatment fields (mm)	
Mean	11.8 (SD ±7.4)

^†^ PI-RADS, Prostate Imaging and Reporting and Data System.

^‡^ ISUP, International Society of Urologic Pathologists.
